# Low-Dose IL-2 Therapy in Autoimmune and Rheumatic Diseases

**DOI:** 10.3389/fimmu.2021.648408

**Published:** 2021-04-01

**Authors:** Hanna Graßhoff, Sara Comdühr, Luisa R. Monne, Antje Müller, Peter Lamprecht, Gabriela Riemekasten, Jens Y. Humrich

**Affiliations:** Department of Rheumatology and Clinical Immunology, University Hospital Schleswig-Holstein Lübeck, Lübeck, Germany

**Keywords:** interleukin-2, immunotherapy, immune regulation, immune tolerance, regulatory T cell, autoimmunity, inflammation

## Abstract

Regulatory T cells (Treg) are crucial for the maintenance of peripheral tolerance and for the control of ongoing inflammation and autoimmunity. The cytokine interleukin-2 (IL-2) is essentially required for the growth and survival of Treg in the peripheral lymphatic tissues and thus plays a vital role in the biology of Treg. Most autoimmune and rheumatic diseases exhibit disturbances in Treg biology either at a numerical or functional level resulting in an imbalance between protective and pathogenic immune cells. In addition, in some autoimmune diseases, a relative deficiency of IL-2 develops during disease pathogenesis leading to a disturbance of Treg homeostasis, which further amplifies the vicious cycle of tolerance breach and chronic inflammation. Low-dose IL-2 therapy aims either to compensate for this IL-2 deficiency to restore a physiological state or to strengthen the Treg population in order to be more effective in counter-regulating inflammation while avoiding global immunosuppression. Here we highlight key findings and summarize recent advances in the clinical translation of low-dose IL-2 therapy for the treatment of autoimmune and rheumatic diseases.

## Introduction

Since the 1970s, when the cytokine interleukin-2 (IL 2) was first discovered and cloned, the view on its function and role in the immune system has changed fundamentally (1, 2). Because of its property to promote the proliferation of T cells *in vitro*, IL-2 was originally considered a key factor for the induction of inflammatory immune responses against invading pathogens and tumors and was therefore introduced in a high dose setting to treat malignant diseases (1, 3). A crucial finding scrutinizing the initial view on IL-2 was that, instead of the expected immune deficiency, mice genetically deficient for IL-2 or IL-2 receptor components developed generalized and fatal autoimmune syndromes due to an uncontrolled hyperactivity of T and B cells (4–8). Later studies could clearly link IL-2 with immune tolerance by showing that IL-2 is essentially required for the growth and survival of regulatory T cells (Treg) in the peripheral lymphatic organs and for their thymic development and differentiation, highlighting the fundamental importance of IL-2 in Treg biology (9–11). Thus, nowadays, IL-2 should rather be considered an “immune regulatory” cytokine and may be by far less important than previously anticipated for the generation of pro-inflammatory and anti-tumor immune responses (8). This novel and scientifically substantiated perception paved the way for the therapeutic exploration of the Treg-IL-2 axis in the setting of immune-mediated and inflammatory diseases with the aim to expand the Treg population directly in the patient thereby counteracting pathogenic autoimmune responses and re-establishing immune tolerance. In later studies, apart from its central role in Treg biology, IL-2 was also shown to inhibit germinal center formation and autoantibody generation by limiting the differentiation of T follicular helper (Tfh) cells independent of Treg interference (12, 13) and to constrain the differentiation of naïve helper T cells into Th17 cells (14). These CD4+ T cell subsets are considered to play a pathogenic role in a large variety of autoimmune and rheumatic diseases.

The principle of using low doses of IL-2 for the treatment of immunological diseases, instead of high doses as long time approved for cancer therapy, was introduced because of the assumption, and meanwhile convincingly proven fact, that Treg are more sensitive to IL-2 and require by far much lower doses of IL-2 for their stimulation compared to anti-tumor T cells and NK cells, because they constitutively express high levels of the heterotrimeric high affinity IL-2 receptor complex which is composed of CD25 (α-chain), CD122 (β-chain) and CD132 (common γ-chain) (3, 8, 15, 16). By comparison, CD4+ conventional T cells (Tcon), CD8+ T cells or NK cells usually express the trimeric IL-2 receptor only upon robust activation (e.g. ligation of TCR). In addition, the acceptance of severe toxicities and side effects associated with high-dose IL-2 therapy seemed not to be justifiable in non-malignant conditions such as autoimmune diseases. Up to now most clinical studies used the human recombinant IL-2 analogue aldesleukin, which has a similar biological activity and a nearly identical biochemical structure than the native human IL-2 protein.

## Rationales for IL-2 Therapy in Autoimmune and Rheumatic Diseases

Treg that express the lineage specific transcription factor FoxP3 are indispensable for the maintenance of immunological self-tolerance and thus for the prevention and control of autoimmune diseases (15, 17–20). Predominantly derived from a distinct CD4+ T cell subpopulation in the thymus, FoxP3+ Treg principally recognize auto-antigens and are required to control the activation, differentiation and expansion of auto-reactive T cells and other potentially harmful immune cells in the peripheral lymphatic organs (15, 21, 22). Consequently, it is reasonable to assume that a disturbance of Treg biology either at a numeric or functional level is involved in the pathogenesis of most rheumatic and autoimmune diseases (18, 23). Apart from this, the survival and growth of Treg fundamentally depend on the availability of IL-2, constituting a vulnerable point in Treg biology (9, 10), and a relative deficiency or shortage of IL-2 can develop in autoimmune diseases leading to a disturbance of Treg homeostasis which further amplifies the vicious cycle of tolerance breach and chronic inflammation (24–26). Low-dose IL-2 therapy aims either to compensate for this shortage of IL-2 or to strengthen the Treg population in order to be more effective in counter-regulating inflammation while avoiding global immunosuppression (16, 27–29).

From an immune-pathophysiological point of view systemic lupus erythematosus (SLE) appeared to be an ideal and promising candidate disease for a therapeutic intervention by low-dose IL-2 therapy. SLE is a prototypic systemic autoimmune disease of unknown etiology characterized by tolerance breach to a large variety of nuclear auto-antigens leading to inflammation in multiple organs (30, 31). Up to date, numerous studies have investigated the role of Treg in mouse models of lupus and in SLE patients and based on these findings it is meanwhile broadly accepted that a disturbance in Treg biology, in particular of the Treg-IL-2 axis, plays a significant role in the pathogenesis of this complex disease (24, 25, 27, 32–38). As early as in the 1980s, a long time before the era of Treg, T cells from SLE patients and lupus-prone mice were found to be impaired in their production of IL-2 upon activation (39–41). Although the pathophysiological relevance of this finding was unclear at this time, following studies demonstrated a recovery from autoimmunity in the MRL/lpr lupus mouse model after vaccination with an IL-2 expressing recombinant vaccinia virus, providing the first evidence that IL-2 therapy could be an effective treatment for SLE (42). Nearly two decades later, a causal relationship between an acquired and progressive deficiency of IL-2 and a disturbance of Treg homeostasis could be identified in the (NZBxNZW) F1 mouse of SLE (24). This self-amplifying disruption of the Treg-IL-2 axis promoted the hyperactivity of pathogenic Th1 cells and accelerated disease progression. Treatment of these mice with IL-2 increased numbers and frequencies of FoxP3+CD25+ Treg and ameliorated ongoing disease (24). In analogy to murine lupus, also human SLE patients were found to exhibit typical signs of IL-2 deficiency, which were characterized by the loss of the CD25hi expressing Treg subset and an imbalanced proliferation between Treg and Tcon, together causing an insufficient availability and exhaustion of highly suppressive and metabolically competent Treg (25). These Treg defects were associated with disease severity and could be corrected *in vitro* and *in vivo* by short-term stimulation with low doses of IL-2, indicating the reversibility of these Treg defects (25). Of note, the *in vitro* suppressive function of Treg from SLE patients was not impaired suggesting that expansion of the endogenous Treg population by low-dose IL-2 therapy is a feasible approach to strengthen immune tolerance. Together, these studies demonstrated the pathophysiological importance of a disturbed Treg-IL-2 axis in SLE and constituted the scientific basis for the clinical introduction of low-dose IL-2 therapy in SLE. In addition to this, it was shown that Tfh cells are expanded in SLE patients (43, 44), providing a complementary rationale for low-dose IL-2 therapy in order to inhibit the differentiation and expansion of T cells, which are required for the generation of autoantibody secreting B and plasma cells (13, 44, 45).

Studies in non-obese diabetic (NOD) mice could demonstrate that a local deficiency of IL-2 in pancreatic islets contributes to the development of type-1 diabetes (T1D), which could be prevented by low-dose administration of IL-2/anti-IL-2 complexes (26). Complementary to this, low-dose IL-2 treatment was also capable to reverse already established murine T1D by promoting the survival and function of Treg (46). These animal studies provided important rationales for the use of low-dose IL-2 therapy in the treatment of this organ specific autoimmune disease.

A lower prevalence of Treg or phenotypic and functional abnormalities of the Treg population have been described also in other rheumatic and autoimmune disease such as rheumatoid arthritis, psoriatic arthritis, ankylosing spondylitis, polymyositis, dermatomyositis, Sjogren’s syndrome and different forms of vasculitis (16, 18, 23, 29). Although the findings here are less clear compared to those in SLE and in part even inconsistent, it appears justified to suppose, in consideration of the immune pathogenesis of these diseases, that expansion of the Treg population or inhibition of Tfh and Th17 cell differentiation by low-dose IL-2 therapy could be a potential treatment option for a large variety of autoimmune and rheumatic diseases.

## Pilot Studies and Clinical Trials

The first pilot studies using low-dose IL-2 therapy in the treatment of human immune-mediated diseases were already started in 2005 and simultaneously published in 2011. Independent from each other, two uncontrolled open-label trials investigated the clinical efficacy and safety of a low-dose IL-2 regimen with aldesleukin in patients with hepatitis C-associated vasculitis and graft-versus-host disease (GvHD), respectively (47, 48). The rationale for conducting these trials was mainly based on the previous finding that numbers and frequencies of CD4+(FoxP3+)CD25+ Treg were reduced in patients with these diseases (49, 50). Both studies demonstrated that repetitive treatment with subcutaneously applied IL-2 at low doses induced an expansion of the CD4+CD25+FoxP3+ Treg population and was effective in reducing clinical symptoms and associated immunological abnormalities. In addition, low-dose IL-2 was well tolerated and most adverse events (AE) were mild and of a transient nature, suggesting a favorable safety profile. To date, since the publication of these encouraging pioneer studies, more than 30 different autoimmune and inflammatory diseases have been treated with low-dose IL-2 therapy in pilot studies, uncontrolled clinical trials and lastly also randomized clinical trials, including SLE, T1D, rheumatoid arthritis, ankylosing spondylitis, psoriasis, Behcet’s disease, granulomatosis with polyangiitis, Takayasu’s disease, Crohn’s disease, ulcerative colitis, autoimmune hepatitis, sclerosing cholangitis, Sjogren´s syndrome, alopecia areata and inflammatory myopathies (see **Table 1** for details) (16).

### Type-1 diabetes (T1D)

Briefly after the publication of the above mentioned pioneer studies, Long et al. conducted a phase I clinical trial investigating a combination therapy with low-dose IL-2 and rapamycin in nine patients with T1D (51). Patients were treated with 2-4 mg/day rapamycin orally for three months and three times per week for one month with 4.5 million IU of subcutaneously administered aldesleukin (12 doses of aldesleukin in total). All treated patients had a biologic response with an increase in numbers and frequencies of CD4+CD25+CD127lo Treg. In parallel, transient increases in numbers of NK cells and eosinophils, but no increases in effector T cells were observed. However, despite the favorable biologic response, all treated patients developed a transient impairment of pancreatic β-cell function. It was suspected that the negative effect on β-cell function was related to the concomitant treatment with rapamycin rather than to IL-2. Reported AEs consisted of mild to moderate fatigue, malaise and injection site reactions.

Regardless of this initial obstacle, one uncontrolled and two randomized placebo-controlled phase I/II trials, one in adults and one in children with T1D (**Table 1**), have been conducted in recent years (52–55). The first randomized, placebo-controlled trial was a single-center, dose-finding trial, that evaluated the safety and the biological efficacy of low-dose IL-2 therapy in 24 adult patients with established T1D. Patients received subcutaneously applied IL-2 (aldesleukin) at single daily doses either of 0.33, 1.0 or 3.0 million IU or placebo (6 patients in each group) for 5 consecutive days (52, 53). Patients were followed-up for 60 days after the 5-day treatment course. Low-dose IL-2 therapy was well tolerated at all applied single doses. AEs were generally mild to moderate (grade 1-2) and resolved spontaneously or with symptomatic treatment. However there was an association between the applied dose and the occurrence of AEs. The most frequently observed treatment-related AEs were pain and erythema at injection sites, fever and influenza-like symptoms. A recently published multi-center, randomized, double-blind, placebo-controlled, dose-finding phase I/II study in 24 children with newly diagnosed T1D confirmed the very good tolerability and safety profile of low-dose IL-2 therapy also in children (transient and mild to moderate AEs, most frequently injection site reactions). In addition, an improved maintenance of induced C-peptide production after one year of treatment was observed in patients who had an increase in Treg of at least 60% after the 5-day induction period. A biological response in form of an effective expansion of the Treg population could be reliably demonstrated in all studies (**Table 1**). Of note, in none of these trials a negative effect on diabetes control, insulin requirements or β-cell function could be observed. This remarkable difference to the study of Long et al. could be due to differences in administered single or cumulative dose of IL-2. Moreover, *in vivo* experiments in NOD mice revealed that rapamycin is cytotoxic to pancreatic β-cells, increases peripheral insulin resistance and even abrogates IL-2-induced cure of diabetes (70–72).

**Table 1:** Summary of results from clinical studies with low-dose IL-2 therapy in autoimmune and rheumatic diseases.

Condition	Trial phase/study aims	Groups	IL-2 administration	Biological responses	Clinical responses	Safety data	Ref.
**HCV-induced vasculitis**	Single-centre, uncontrolled, phase I/IIa clinical trial: safety, biological efficacy, clinical outcomes	IL-2: n=10	s.c. injections of 1.5 MIU/d for 5 d, followed by three 5-d courses of 3 MIU/d at weeks 3, 6, and 9 (9 weeks)	%T_reg_ (CD4^+^CD25^hi^CD127^lo^FOXP3^+^) ↑, % CD8^+^ (CD8^+^CD25^+^FOXP3^+^) T_reg_ ↑, ratio T_reg/_T_con_ ↑, T_reg_ supp. =, NK cells/µl ↑, CD56^bright^ NK cells/µl ↑, CD19^+^ B cells/µl ↓, CD19+IgD+CD27+ B cells/µl ↓, transcriptome: T_reg_ and NK cell function related ↑, inflammatory and oxidative stress mediators ↓	improvement in vasculitis in 8 of 10 patients, decrease in cryoglobulins in 7 of 10 patients, complement C4 ↑, modest decrease in HCV viral load	SAE: Ø; TR-AE: injection-site reaction, asthenia, influenza-like symptoms, myalgia, dental abscess	(47)
**Insulin-dependent type 1 diabetes mellitus**	Single-centre, uncontrolled phase I clinical trial: safety, biological efficacy	IL-2: n=9	s.c. injections of 4.5 MIU/d 3x/week for 4 weeks; loading dose of rapamycin 2 mg/d, followed by dose adjustments to maintain blood levels of 5-10 ng/ml for 3 months	T_reg_/µl (CD3^+^CD4^+^FOXP3^+^) ↑, % T_reg_ (CD4^+^CD25^+^CD127^lo^) ↑, FOXP3 gene demethylation ↑, %IFNg+ among T_reg_ (CD4^+^FOXP3^+^) =, IL-2 responsiveness in T_reg_ (CD4^+^CD25^+^) measured by STAT5 phosphorylation ↑, % CD45RO^-^ and CD45RO^+^ among CD4^+^ and CD8^+^ T cells =, % CXCR3^+^, CRTH2^+^, IFNg^+^ and IL-17^+^ among CD4^+^CD45RO^+^ =, CD56^+^ NK cells/µl ↑, % CD56^bright^ NK cells ↑, eosinophils/µl ↑, lymphocytes/µl =, monocytes/µl =, CD4^+^/CD8^+^ ratio =, sIL-2R↑ (correlation with increase in % T_reg_, NK cells, eosinophils)	transient β-cell dysfunction, peak C-peptide levels from MMTT ↓, stable HbA_1c_ was achieved with increasing doses of insulin	SAE: Ø; TR-AE: injection-site reaction, fatigue, malaise, abdominal pain	(51)
	Single-centre, randomized, placebo-controlled, double-blind, dose-finding, phase I/II clinical trial: safety, biological efficacy, clinical outcomes	n=24, randomized to placebo, or 0.33, 1 or 3 MIU/d IL-2 (1:1:1:1)	s.c. injections of 0·33 MIU, 1 MIU, or 3 MIU/d for 5 consecutive days (1 cycle)	T_reg_/µl and % T_reg_ (CD4^+^CD25^hi^CD127^lo^ FOXP3^+^) ↑ (dd), iEmax and iAUC in %T_reg_ ↑ (dd), iEmax % NK cells and % T_eff_ =, % CD19^+^ B cells ↓, CD19^+^ B cells/µl ↓ (dd), iEmin for change in % CD19^+^ B cells ↓, eosinophils ↑	no significant differences between groups in daily insulin dose, fasting glycaemia, fasting plasma C-peptide and iAUC during an MMTT, HbA_1c_	SAE: Ø; TR-AE: injection-site reaction (dd), influenza-like symptoms (dd), nausea (dd), allergic rhinitis, diarrhea, fatigue, ophthalmic migraine	(52)
	Single-centre, randomized, placebo-controlled, double-blind, dose-finding, phase I/II clinical trial (ref. 52): biological efficacy, immunophenotyping	see ref. 52	see ref. 52	T_reg_/µl and % T_reg_ (CD4^+^CD25^hi^CD127^lo^FOXP3^+^) ↑ (dd), T_reg_/T_eff_ ratio ↑, duration of T_reg_ increase (dd), % CD8^+^CD25^+^Foxp3^+^ T_reg_ ↑ (dd), % CD25^hi^CD127^lo^CD45RA^-^ T_reg_ of CD4^+^ ↑ (dd), CD25 (MFI), GITR, CTLA-4 and basal pSTAT5 in T_reg_ ↑ (dd), IL-2 responsiveness in T_reg_ (CD4^+^FOXP3^+^) measured by STAT5 phosphorylation =, counts lymphocytes, CD4^+^, CD8^+^ T cells, NK cells during cycle ↓ after cycle ↑ (dd), % CD4^+^, CD8^+^ T cells =, % CD19^+^ B-cells and CD19^+^ B cells/µl ↓ (dd), % NK cells ↑ (dd), % CD56^bright^ among NK cells ↑ (dd), plasma levels of IL-2, sIL-2R, IL-5, IL-10, IL-17, TNF-a, TGF-b1, CCL22, CXCL10 ↑ (dd), transcriptome: B cell related ↓, cell cycle and transcription ↑, chemokine and NK cell related ↑ (dd), FOXP3 target genes ↑ (dd), suppression of T_eff_ cell responses (IFNg) against beta-cell antigens (dd); placebo group: no relevant changes	see ref. 52	no detection of anti-IL-2-antibodies; see also ref. 52	(53)
	Single centre, uncontrolled, adaptive dose-finding phase, I/II clinical trial: safety, biological efficacy, acute cellular responses to different single doses	Learning phase: n=10; adaptive phase: n=30	learning phase: s.c. injections of one single dose per patient either of 0.004 (n=2), 0.16 (n=2), 0.60 (n=2), 1.00 (n=2), or 1.50 (n=2) MIU/m^2^ in ascending order; adaptive phase: s.c injections of one single dose per patient. based on dose-finding results of interim analyses to achieve T_reg_ increases of 10% and 20% from baseline	T_reg_/µl and % T_reg_ (CD3^+^CD4^+^CD25^hi^CD127^lo^) ↑ (dd) peak by d2/3 (d1 ↓); IL-2 doses to induce 10%/20% increases in T_reg_: 0.101 MIU/m^2^ and 0.497 MIU/m^2^, IL-2 plasma levels ↑ (peak at 90 min, dd), MFI CD25, pSTAT5, CTLA-4, FOXP3, CXCR3, CCR6, % Ki67^+^ of CD45RA^-^ memory T_reg_ ↑ (dd, peaks between 90 min. and d2), CD122 MFI in memory T_reg_ ↓ (dd, peak at 90 min), FOXP3 demethylation and T_reg_ supp. =, sIL-2R ↑ (dd), MFI CD25, CD122 ↓, MFI pSTAT5, % Ki67 ↑ of CD45RA^-^ memory T_con_ (dd), lymphocytes/µl ↓ (dd), neutrophils/µl ↓, CD8^+^ T cells/µl, CD19^+^ B cells/µl, NK cells/µl ↓ (dd, recovering to baseline by d4); eosinophils/µl ↓ at 90 min, followed by increase with peak by d1 (dd), % CD56^bright^ NK cells ↑ (at 90 min ↓), MFI pSTAT5, %Ki67 of CD56^bright^ NK cells ↑	Mean glucose ↓, HbA_1c_ ↓, basal insulin dose ↓, non-fasting C-peptide =	SAE: Ø TR-AE: injection-site reaction, common cold, nasal congestion, CRP ↑ (dd, peak by d1)	(54)
	Multicentre, randomized, double blind, placebo-controlled, dose-finding phase I/II clinical trial in children: safety, biological efficacy, clinical efficacy	n=24, randomized to placebo or 0.125, 0.250, 0.500 MIU/m^2^ IL-2 (7:5:6:6)	s.c. injections of placebo or IL-2 at doses of 0.125, 0.25 or 0.5 MIU/m^2^ daily for five days and then fortnightly for 1 year	% T_reg_ (CD4^+^CD25^hi^CD127^lo^FOXP3^+^) ↑ (dd), T_reg_/T_eff_ ratio ↑, maintenance of T_reg_ response with 2 highest doses, CD25^+^ T_eff_ =, B cells =, NK cells =, sIL-2RA and VEGFR2 levels predicted T_reg_ response after the 5-day course; eosinophils ↑; placebo group: no relevant changes	no significant change between IL-2 and control group in C-peptide iAUC in MMTT, HbA_1c_, fasting blood glucose, fasting C-peptide level, insulin requirements. In patients with T_reg_ increase > 60% from baseline (d5): improved maintenance of induced C-peptide production at 1 year	SAE: Ø; TR-AE: injection-site reactions	(55)
**Systemic lupus erythematosus**	Pilot study / compassionate use in refractory SLE: safety, biological efficacy, clinical outcomes	IL-2 + SOC: n=1	four cycles with daily s.c. injections between 1.5 and 3.0 MIU/d for 5 d; separated by resting periods of 9-16 d (9 weeks)	T_reg_/µl and % T_reg_ during cycles (CD3^+^CD4^+^Foxp3^+^CD127^lo^CD25^hi^) ↑	decrease in SLEDAI from 14 to 4 after 1st cycle, no development of new organ manifestations/disease flares during treatment, reduction of daily GC dose, decrease in anti-dsDNA-Abs, normalization of CK	SAE: Ø; TR-AE: injection-site reaction, increased day and night sweats, transient fever	(56)
	Single-centre, uncontrolled phase I/IIa clinical trial: biological efficacy of short-term treatment (immuno-phenotyping data of first 5-day cycle from first 5 patients of PRO-IMMUN trial)	IL-2 + SOC: n=5	s.c. injections of 1.5 MIU/d for 5d (1 cycle)	T_reg_/µl and % T_reg_ (CD3^+^CD4^+^Foxp3^+^CD127^lo^) ↑; % CD25^hi^ of T_reg_ ↑; MFI CD25 in T_reg_ ↑; % Ki67^+^ of T_reg_ ↑, % CD39^+^ of T_reg_ ↑, % Helios^+^ of T_reg_ =, Treg/Tcon proliferation ratio ↑ in 4/5 patients, CD3^+^CD4^+^ T_con_/µl =, CD25 MFI in CD3^+^CD4^+^ T_con_ =; % CD25^hi^ of CD3^+^CD4^+^ T_con_ ↑; % Ki67^+^ of CD3^+^CD4^+^ T_con_ (=↑), % Ki67^+^ of CD8^+^ T cells, NKT cells, NK cells, CD56^bright^ NK cells ↑; counts and % CD8^+^ cells, NK T cells, NK cells =; % CD56^bright^ of NK cells ↑	not evaluated	not evaluated	(25)
	Single-centre, uncontrolled phase I/IIa clinical trial: biological efficacy (immuno-phenotyping data from 23 patients), clinical outcomes	IL-2 + SOC: n=38	three cycles of s.c. injections of 1 MIU every other day for 2 weeks followed by a 2-week break in treatment (10 weeks)	% T_reg_ (CD3^+^CD4^+^CD25^hi^CD127^lo^) ↑, T_reg_ supp. ↑, % T_FH_ of total CD4+ (CD4^+^CXCR5^+^PD-1^+^CCR7^-^) ↓, % T_H_17-like of total CD4+ (CCR6^+^CXCR3^−^CCR4^+^CCR7^-^) ↓, (T_FH_ + T_H_17) cells/T_reg_ cells ↓, % CD3^+^CD4^−^CD8^−^αβ T cells ↓, % T_H_1-like (CXCR3^+^CCR6^−^CCR4^−^CCR7^-^) =, % T_H_2-like (CXCR3^−^CCR6^−^CCR4^+^CCR7^-^) =, eosinophils ↑	SRI-4 response rates: 31.6%/71.1%/ 89.5% at week 2/4/12; GC dose ↓; improvement/resolution in rash, alopecia, arthritis, fever, serositis; resolution of leukopenia/ thrombopenia in 94.7/100% of patients, complement C3 and C4 ↑; anti-dsDNA-Abs ↓, proteinuria ↓	SAE: Ø; TR-AE: injection-site reaction, influenza-like symptoms, very low frequency of total AEs (7 AEs in 38 pat.), total IgG ↓	(57)
	Single-centre, uncontrolled, dose-adaption phase I/IIa clinical trial in refractory SLE (PRO-IMMUN): safety, tolerability, biological efficacy, dose-dependency of biological responses, clinical outcomes	IL-2 + SOC: n=12	four cycles with s.c. injections between 0.75 and 3.0 MIU/d for 5 d separated by resting periods of 9-16 d (9 weeks)	T_reg_ /µl and % T_reg_ (CD3^+^CD4^+^FOXP3^+^CD127^lo^) ↑ (dd),% CD25^hi^ of T_reg_ ↑ (dd), % CD25^hi^ T_reg_ of CD3^+^CD4^+^ ↑, (dd) CD25^hi^ T_reg_/µl ↑ (dd), MFI CD25 in T_reg_ ↑ (dd), % Ki67^+^ of T_reg_ ↑ (dd), ratio counts T_reg_/T_con_ ↑, ratio Ki67^+^ T_reg_/Ki67^+^ T_con_ ↑; corr. increase % CD25^hi^ T_reg_ with increase % Ki67^+^ T_reg_, % CD39^+^ of T_reg_ ↑, % Helios^+^ of T_reg_ =, % CD137^+^ of T_reg_ ↑; T_reg_ supp. and FOXP3 demethylation =, higher % of CD25^hi^ and CD137^+^ T_reg_ in responders after 4 cycles, corr. increase % CD25^hi^ T_reg_ with decrease in SLEDAI, % CD45RO^-^CCR7^+^ of T_reg_ ↓ (during cycles), % CD45RO^+^CCR7^+/-^ of T_reg_ =, % CXCR5^+^ of T_reg_ (TfR) ↓, sIL-2R ↑ (dd), corr. increase sIL-2R and GC dose with increase % CD25^hi^ T_reg_ and increase % Ki67^+^ T_reg_; T_con_/µl (CD3^+^CD4^+^FOXP3^-^) =, % CD25^hi^ in T_con_ = (at highest dose ↑), MFI CD25 in T_con_ =, % Ki67^+^ of T_con_ ↑ (dd), % CD45RO^+/-^CCR7^+/-^ T_con_ subsets =, % CD3^+^CD4^-^CD8^-^ T cells =, % T_FH_ of T_con_ (CXCR5^+^ and CD45RO^+^CCR7^-^CXCR5^+^PD-1^+^) ↓, % T_H_17-like of T_con_ (CD45RO^+^CCR7^-^CCR6^+^CXCR3^−^CCR4^+^) =; CD19^+^ B cells/µl ↓, counts and % CD19^+^ IgD^+^CD27^+^ B cells ↓, counts CD19^+^ IgD^-^CD27^+^ and CD19^+^ IgD^-^CD27^-^ ↓, counts and % of CD19^+^ CD20^-^CD27^hi^HLA-DR^+/-^ =; counts CD8^+^ cells, NK T cells, NK cells =, % Ki67^+^ of CD8^+^ T cells, NK T cells, NK cells ↑ (dd), eosinophils ↑	Decrease in SLEDAI score/clinical response in 83%/67% of patients at day 62 (significant decrease already after 2^nd^ cycle), improvement / resolution in arthritis, myositis, rash, alopecia; no severe disease flare; PGA ↓; frequency of BILAG severity categories A and B ↓; complement C3 ↑ (during cycles); anti-dsDNA-Abs =	SAE: Ø (5 unrelated SAE in FU-phase); TR-AE (dd): injection-site reactions, fever and chills, influenza-like symptoms, headache, dizziness, arthralgia, myalgia; transient increases in CRP (dd), D-dimers and other acute-phase proteins without clinical relevance during cycles (complete normalization in resting phases); ECG, abdominal ultrasound, echocardiography, lung function =	(58)
	Single-centre, open-label, controlled phase I/II clinical trial in lupus nephritis: safety, biological efficacy, clinical outcomes	IL-2 + SOC: n=18, SOC: n=12	3 cycles of s.c. injections of 1 MIU every other day for 2 weeks followed by a 2-week break in treatment (10 weeks)	% T_reg_ (CD3^+^CD4^+^CD25^hi^CD127^lo^) ↑, stronger increase of % T_reg_ in patients who achieved remission; sIL-2R (sCD25) =; control group: no relevant changes	Higher remission rate in IL-2 group compared to SOC at week 10: 55.6% vs 16.7%, p=0.058; improved renal outcomes in IL-2 group at week 10 compared to baseline: 24-h UPE ↓, hematuria↓, albumin (s) ↑, leukocyturia =, urea nitrogen (s) =, creatinine (s) =, eGFR =	SAE: Ø; TR-AE: injection-site reaction, fever, influenza-like symptoms, nausea, and diarrhea	(59)
	Single-centre, uncontrolled phase I/II clinical trial in refractory SLE: biological efficacy, clinical outcomes	IL-2 + rapamycin: n=50	s.c. injection of 100 WIU 3-5d/months combined with rapamycin (0.5 mg, once every other day, oral) for 24 weeks	T_reg_/µl (CD4^+^CD25^+^FOXP3^+^) ↑ at week 12 and 24, T_H_17 cells/µl (CD4^+^ IL17^+^) =, ratio T_H_17/T_reg_ ↓ at week 24	Decrease in SLEDAI score after 6 (p=0.002), 12 (p<0.0001), 24 weeks (p<0.0001) compared to baseline; prednisone dose ↓; DMARD dose =	SAE: Ø; TR-AE: not evaluated	(60)
	Single-centre, randomized, double-blind, placebo-controlled phase II clinical trial: safety, clinical efficacy	IL-2 + SOC: n=30 placebo+SOC: n=30	3 cycles of s.c. injections of 1 MIU every other day for 2 weeks followed by a 2-week break in treatment (10 weeks)	IL-2 group: %T_reg_ (CD3^+^CD4^+^CD25^hi^CD127^lo^) ↑; % NK cells ↑, % CD56^bright^ of NK cells ↑, % CD3^+^CD4^+^ and CD3^+^CD8^+^ T cells =; placebo group: no relevant changes	SRI-4 response rates: 55.17%/65.52% in IL-2-group vs 30.00%/36.67% in placebo group at week 12 (p=0.052) and week 24 (p=0.027): primary endpoint at week 12 not met;no sign. difference between IL-2 and placebo group in change of SLEDAI, BILAG, PGA, and prednisone dose; higher improvement rate for rash and arthritis in IL-2 group, complete remission in pat. with lupus nephritis in 53.85% in IL-2 group vs 8.33% at week 12 (p=0.013) and 16.67% at week 24 (p=0.036) in placebo group; 24-h UPE ↓, (s)albumin ↑, complement C3/C4 ↑ (ns), anti-dsDNA-abs ↓ in IL-2 group compared to baseline	SAE: Ø; TR-AE: injection-site reaction, influenza-like symptoms, fever; lower incidence of infections in IL-2 group than in placebo group	(61)
	Randomized, double-blind, placebo-controlled, multiple ascending dose phase Ib clinical trial: safety, biological efficacy	NKTR-358 3.0 µg/kg (n=9), 6.0 µg/kg (n=9), 12.0 µg/kg (n=9), 24.0 µg/kg (n=9); placebo: n=12	s.c. injections of NKTR-358 at 3.0 µg/kg, 6.0 µg/kg, 12.0 µg/kg, 24.0 µg/kg once every 2 weeks, 3 times in total (4 weeks)	T_reg_/µl and % T_reg_ (CD4^+^FOXP3^+^CD25^hi^) ↑ (dd), % Ki67^+^ of T_reg_ ↑ (dd), FOXP3 demethylation ↑, expression of CD25, Helios, CTLA-4 in T_reg_ ↑ (dd), CD56^+^ NK cells ↑ (dd), CD56^bright^ NK subset ↑ (dd), CD4^+^ and CD8^+^ T cells =; placebo group: no relevant changes	Reduction of CLASI-A score ≥ 4 points compared to baseline at day 43 in 7/18 patients; SLEDAI =; joint scores =	SAE: Ø; TR-AE: injection-site reaction, influenza-like symptoms, eosinophilia; no detection of anti-IL-2-antibodies	(62)
**Alopecia areata**	Single-centre uncontrolled phase I clinical trial in refractory disease: biological efficacy, clinical outcomes	IL-2: n=5	s.c. injections of 1.5 MIU/d for 5 d, followed by three 5-d courses of 3 MIU/d at weeks 3, 6, and 9 (9 weeks)	T_reg_/µl (CD3^+^CD4^+^FOXP3^+^CD127^lo^CD25^+^) ↑ (ns); skin biopsies: T_reg_ ↑ in 4/5 patients, CD8^+^ T cells ↓, persistent T_reg_ increase 2 months after end of treatment	Regrowth of scalp hair in 4/5 patients, continuation of improvement up to 6 months; median SALT scores 2/6 months after end of treatment: 76/69 (Baseline: 82); DLQI ↓	SAE: Ø; TR-AE: asthenia, arthralgia, urticaria, injection-site reaction	(63)
**Autoimmune hepatitis**	Pilot study / compassionate use in refractory disease: biological efficacy, clinical outcomes	IL-2: n=2	6 monthly cycles of s.c. injections of 1 MIU for 5d (6 month)	% T_reg_ (CD4^+^FOXP3^+^CD25^+^) ↑, % CD45RA^+^FOXP3^lo^ and CD45RA^-^FOXP3^hi^ ↑, % Ki67^+^ of T_reg_ ↑, MFI CD25 and FOXP3 ↑, sIL-2R ↑; % CD4^+^ T_con_ and NK cells ↓	Normalization of liver enzymes and serum levels of IgG in 1 patient	Not evaluated	(64)
**RA, AS, SLE, psoriasis, Behçet’s disease, GPA, Takayasu’s disease, CD, UC, AIH, sclerosing cholangitis**	Multicentre, uncontrolled phase I/IIa clinical basket trial in 11 autoimmune diseases (TRANSREG): safety, biological efficacy, disease selection	RA (n=4), AS (n=10), SLE (n=6), psoriasis (n=5), Behçet’s disease (n=2), GPA (n=1), Takayasu’s disease (n=1), CD (n=7), UC (n=4), AIH (n=2), sclerosing cholangitis (n=4) (in total 46 patients)	induction phase: s.c. injections of 1 MIU/d for 5 d; maintenance phase: fortnightly injections of 1 MIU/d for 6 months	T_reg_/µl and % T_reg_ (CD4^+^FOXP3^+^CD127^lo^CD25^hi^) ↑ (peak at d8), AUC %T_reg_ ↑, T_con_ (FOXP3^−^ CD4^+^ and CD8^+^ cells) =, activated T_eff_ (CD4^+^CD25^lo/+^FOXP3^−^) =, T_reg_/T_eff_ ratio ↑, T_reg_/activated CD4^+^ T_con_↑, counts and % CD3^+^, CD4^+^ T cells, NK cells = (↑ at d8), counts and % CD8^+^ T cells, CD19^+^ B cells = (% ↓ at d8), =, % CD56^bright^ of NK cells ↑, eosinophils ↑ (transient), plasma levels of T_H_1/T_H_2/T_H_17/T_reg_ cytokines =, similar biological efficacy (T_reg_ expansion) across all 11 diseases, no differences due to different background therapies; transcriptome: FOXP3, IL-2R, CTLA-4 genes and related pathways ↑, T_reg_ signature genes ↑	Significant improvement in CGI; improvement in disease-specific scores (AS, UC, SLE, psoriasis); % of patients with fatigue and arthralgia ↓; improvement in EuroQL-5D-5L-score (ns),	SAE: Ø (7 unrelated SAE); TR-AE: injection-site reaction, seasonal upper and lower respiratory tract infections, no detection of anti-IL-2-antibodies	(65)
**Primary Sjögren’s syndrome**	Single-centre, open-label, controlled phase I/II clinical trial: biological efficacy of short-term treatment	IL-2 + SOC: n=99, SOC: n=91	s.c. injections of 0.5 MIU/d for 5d (1 cycle)	T_reg_/µl (CD4^+^CD25^+^FOXP3^+^) ↑, T_H_17 cells/µl ↑, T_H_17/T_reg_ ratio ↓; control group: no relevant changes	no difference in disease activity between IL-2 and control group; glucocorticoid and DMARDs usage ↓ (long-term)	SAE: Ø; TR-AE: injection-site reactions, influenza-like symptoms,	(66)
**Polymyositis/** **Dermatomyositis**	Single-centre, open-label, controlled phase I/II clinical trial: biological efficacy of short-term treatment	IL-2 + SOC: n=31, SOC: n=116	s.c. injections of 0.5 MIU/d for 5d (1 cycle)	T_reg_/µl ↑, counts T cells, B cells, CD4^+^ T cells, CD8^+^ T cells ↑, T_H_1 ↑, T_H_2 ↑, T_H_17 ↑, NK cells =; (gating strategy / definition of subsets not provided); control group: no relevant changes	VAS, ESR, CK, CK-MB, LDH, HBDH ↓ in IL-2 and control group compared to baseline, VAS ↓ in IL-2 group compared to control group (short-term)	not evaluated	(67)
**Psoriatic arthritis**	Single-centre, uncontrolled phase I/II clinical trial: safety, biological efficacy, clinical outcomes of short-term treatment	IL-2 + SOC: n=22	s.c. injections of 0.5 MIU/d for 5 d (1 cycle)	T_reg_/µl and % ↑, T_H_17/µl ↑, ratio T_H_17/T_reg_ = , T_H_1/µl =, T_H_2/µl =, ratio T_H_1/T_H_2 =; % T_H_17 cells ↑, % T_H_1 =, % T_H_2 = ; (gating strategy / definition of subsets not provided)	TJC, SJC, VAS, ESR, DAS28-ESR, PGA, DLQI, HAQ ↓ in IL-2 group compared to baseline (short-term)	SAE: Ø; TR-AE: injection-site reaction	(68)
**Amyotrophic lateral sclerosis**	Single centre, parallel three-arm, randomized, double-blind, placebo-controlled phase IIa clinical trial: safety, biological efficacy, clinical outcomes	IL-2 + riluzole: 1 MIU (n=12), 2 MIU (n=12),; placebo + riluzole: n=12	3 cycles with s.c. injections of 1 or 2 MIU/d or placebo for 5 d every 4 weeks (9 weeks)	T_reg_/µl and % T_reg_ (CD4^+^FOXP3^+^CD127^-^CD25^hi^ ) ↑ (dd), T_reg_ supp. ↑, % NK cells ↑, % CD8^+^ T cells ↑, % CD4^+^T_con_ ↑, % monocytes ↓, MFI CD25 in T_reg_ ↑, MFI CD25 in CD4^+^ T_con_ ↑, eosinophils ↑, plasma levels of CCL2 ↓ (dd), CCL17 and CCL18↑ (dd),; placebo group: no relevant changes	No significant differences in disease progression among the three groups regarding ALSFRS-R score, decline in vital capacity and plasma NFL-MSD levels	SAE: Ø; TR-AE: injection-site reaction (dd), influenza-like symptoms(dd), nausea/vomiting, urinary retention	(69)

Abs, antibodies; AIH, autoimmune hepatitis; ALSFRS-R score, Amyotrophic lateral sclerosis Functional Rating Scale - Revised; AS, ankylosing spondylitis; AUC, area under the curve; BILAG, British Isles Lupus Assessment Group; CD, Crohn’s disease; CGI, Clinical Global Impression Scale; CLASI-A, Cutaneous Lupus Erythematosus Disease Area and Severity; CK, Creatine kinase; d, days; Corr., correlation; DAS, disease activity score; dd, dose-dependent effect; DLQI index, Dermatology Life Quality Index; DMARD, disease-modifying anti-rheumatic drug; eGFR, estimated glomerular filtration rate; ESR, erythrocyte sedimentation rate; GC, glucocorticosteroids; GPA, granulomatosis with polyangiitis; HBDH, α-hydroxybutyrate dehydrogenase; HC, healthy controls; HCQ, hydroxychloroquine; HCV, hepatitis C virus; HAQ, Health Assessment Questionnaire; iAUC, incremental area under the curve; iEmax, incremental maximum effect; iEmin, maximum decrease below baseline; LDH, lactate dehydrogenase; MFI, mean fluorescence intensity; MIU, million International Units; MMTT, mixed meal tolerance test; NFL, neurofilament light chain; NK cells, natural killer cells; NKT cells, natural killer T cells; ns, not significant; PGA, Physician’s Global Assessment; RA, rheumatoid arthritis; s, serum; SAE, serious adverse event; SALT, severity of alopecia tool; SLEDAI, Systemic Lupus Erythematosus Disease Activity Index ; sIL-2R, soluble interleukin-2 receptor; SJC, swollen joint count; SLE, systemic lupus erythematosus; SRI-4, SLE Responder Index-4; SOC, standard-of-care treatment; T_eff_, effector T cells; TJC, tender joint count; TR-AE, treatment-related adverse event; T_reg_, regulatory T cells; T_reg_ supp., in vitro suppressive function of T_reg_; UC, ulcerative colitis; VAS, visual analogue scale; VEGFR2, vascular endothelial growth factor receptor 2; 24-h UPE, 24 hour urine protein excretion.

### Systemic Lupus Erythematosus (SLE)

In 2013, the first SLE patient worldwide was successfully treated with four cycles of low-dose IL-2 therapy in a compassionate use setting (56). The clinical response was accompanied by remarkable increases in numbers and frequencies of the CD4+FoxP3+CD127loCD25hi Treg subset. First results from an phase I/II trial (PRO-IMMUN) demonstrated that one 5-day course of low-dose IL-2 therapy with daily injections of 1.5 million IU was capable to selectively increase CD25 expression in CD4+Foxp3+CD127lo Treg and to promote the efficient and selective expansion of the CD4+FoxP3+CD127lo Treg population in five patients with active SLE (25). Apart from this, moderate increases in the numbers of NK cells, especially of the CD56bright subset could be observed during this short-term treatment. Subsequently, He et al. reported immunological findings from 23 patients obtained during an uncontrolled, single-center study. They found that, in parallel to an increase in the percentage of the CD4+CD25+CD127lo Treg population, low-dose IL-2 therapy led to decreases in the percentages of Tfh and Th17-like cells among total CD4+ T cells (effects on absolute numbers of these subsets have not been provided) (57). Based on the results of these pilot studies, six phase I/II trials have been conducted in recent years including in total app. 300 SLE patients with different clinical manifestations. Administered dose, treatment regimens and treatment duration as well as follow-up-times varied between these clinical trials (**Table 1**).

The first larger clinical trial in Europe started in march 2014 and was a single-center, uncontrolled, dose-adaption, phase I/IIa trial (PRO-IMMUN) with the primary aim to investigate the safety, tolerability and biological efficacy of low-dose IL-2 therapy in 12 patients with active SLE who had refractory disease activity under conventional therapy (58). Patients were treated with four separate cycles of low-dose IL-2 therapy using recombinant human IL-2 (aldesleukin) on top of standard-of-care therapy. Each of the four treatment cycles consisted of daily subcutaneous injections of IL-2 for 5 consecutive days followed by a 9-16 day resting phase in between. The daily dose in the first cycle was 1.5 million IU of IL-2 for all patients. In the subsequent cycles, the single dose was either increased from 1.5 million IU to 3.0 million IU or decreased to 0.75 million IU according to predefined dose adaption and safety criteria. The primary endpoint was the number of patients who achieved at least a 100% increase in the proportion of CD25hi-expressing cells among circulating CD3+CD4+FoxP3+CD127lo Treg at day 62 after four treatment cycles. Secondary study objectives included clinical responses and changes in diverse serological and immunological parameters. The treatment was well-tolerated with single doses of 0.75 and 1.5 million IU and most treatment-related AEs were transient and mild to moderate (grade 1–2). The most frequent AEs were mild injection-site reactions (20% of all AEs). Moderate and transient treatment-related increases in acute-phase proteins, such as C-reactive protein, in the absence of clinically relevant symptoms were noted. Low-dose IL-2 therapy elicited substantial and dose-dependent increases in the proportions and absolute numbers of CD3+CD4+FoxP3+CD127loCD25hi Treg and 11 of the 12 treated patients (92%) achieved the primary endpoint. Apart from moderate and transient increases in the numbers of eosinophils and NK cells, no relevant increases in the numbers of other leukocyte subsets were observed. Low-dose IL-2 therapy also preferentially augmented the proliferation of Foxp3+CD127lo Treg resulting in a partial restoration of the homeostatic balance between Treg and Tcon, which is typically disturbed in SLE patients. Clinical responses were observed in 8 of the 12 treated patients (66·7%) at day 62 after 4 treatment cycles and no severe disease flares occurred during the treatment period. The reduction in disease activity thereby correlated with the magnitude of the Treg response, as measured by the increase in the frequencies of CD25hi Treg among FoxP3+CD127lo Treg. Transient increases in complement levels during the cycles, but no decreases in SLE-associated auto-antibodies were observed. The IL-2 expanded Treg population displayed a preserved suppressive function and demethylated foxp3 locus, maintained high levels of Helios, which is mainly expressed by thymic-derived Treg, and expressed increased levels of the Treg-associated molecules CD39 and CD137. Concomitantly to the efficient and selective expansion of the Treg population, a reduction in numbers of CD19+ B cells, especially of IgD+CD27+ marginal-zone B cells, which was also reported in other diseases (46, 51), and, though less pronounced, in the frequencies of Tfh cells was observed, together suggesting that IL-2 therapy may interfere with the germinal-center reaction in lymphoid organs. This clinical trial proved that low-dose IL-2 therapy safely and selectively promotes the expansion of a functionally competent and thymic-derived Treg population and suggested clinical efficacy of low-dose IL-2 therapy in patients with active and refractory SLE.

Briefly thereafter, the first randomized, placebo-controlled, single-center trial with low-dose IL-2 therapy in 60 patients with active SLE was published (61). In each group, 30 patients received 3 cycles of either low-dose IL-2 at a single dose of 1 million IU or placebo subcutaneously applied every other day for 2 weeks followed by a 2-week break on top of standard treatment. The primary endpoint was the proportion of patients who achieved a SLE responder index-4 (SRI-4) response at week 12 compared to the placebo group. In the IL-2 group 55% of the patients achieved a SRI-4 response at week 12, whereas in the placebo group this was achieved in only 30%. However, although being close to statistical significance, the primary endpoint was not met (p=0.052). Nonetheless, a significant difference in the proportion of patients with a clinical response between the IL-2 and placebo group was observed between week 6 and 10 and during the follow-up phase. In addition, both at week 12 and 24 complete remission was observed in 54% of patients with renal involvement compared to only 8% and 17% in the placebo group (p=0.013 and 0.036). A decrease in anti-dsDNA antibodies was only observed in the IL-2 treated group, and there was a higher percentage of patients who achieved normalization of complement levels. No serious infection occurred in the IL-2 group, but two in placebo group. Similar to previous studies, an expansion of the Treg population and moderate increases in NK cells, especially of the CD56bright subset, were observed in this study.

Complementing these clinical trials, the efficacy of low-dose IL-2 in patients with lupus nephritis was investigated in a single-center, controlled phase I/IIa trial (59). 18 patients received three cycles of low-dose IL-2 on top of standard-of-care treatment, and 12 patients in the control group received standard-of-care treatment only. Consistent with the results from He et al., a higher remission rate in the IL-2-treated group (55%) compared to the control group (17%) (p=0.058) was found after 10 weeks of treatment. In addition, a single-center, uncontrolled study investigated clinical and immunological responses of a combination therapy with low-dose IL-2 applied monthly for 3-5 days and continuous treatment with rapamycin every other day in 50 patients with refractory SLE. The combination therapy was applied for 24 weeks and significantly reduced disease activity and prednisolone dose compared to baseline for up to 24 weeks (60).

Most recently, Fanton et al. reported the biological and clinical effects of IL-2 therapy using four different doses of a novel pegylated IL-2 conjugate named NKTR-358 in SLE patients with cutaneous manifestations (62). Pegylation of IL-2 results in an increase in half-life to approximately 14 days compared to a few hours for subcutaneously applied native IL-2. In patients receiving NKTR-358 a dose-dependent expansion and activation of CD25hi Treg and a reduction in CLASI-A score in 7 of 18 treated patients was noted. Based on these promising results, a phase II clinical trial has already been initiated (NCT04433585).

The results of a meanwhile completed multi-center, randomized, placebo-controlled phase II trial (LUPIL-2) with 50 patients in each group have not yet been published.

### Other Rheumatic and Autoimmune Diseases

As early as in 2014, an open-label pilot trial using low-dose IL-2 therapy in five patients with alopecia areata was published (63). Skin biopsies in four of the five treated patients showed an increase in Treg numbers and a decrease in CD8^+^ T cells, and these patients had a significant improvement in hair regrowth on scalp and body with effects extending beyond treatment period.

Two patients with refractory autoimmune hepatitis had been treated with monthly 5-day cycles of low-dose IL-2 therapy for 6 month in a compassionate use setting (64). In both patients an increase in the frequency of Treg was elicited, and one patient experienced a substantial clinical response with normalization of liver enzymes and total IgG.

More recently an open-label, multi-center phase I/IIa trial in 46 patients with mild to moderate forms of 11 different autoimmune and inflammatory diseases, i.e. rheumatoid arthritis, ankylosing spondylitis, SLE, psoriasis, Behcet’s disease, granulomatosis with polyangiitis, Takayasu’s disease, Crohn’s disease, ulcerative colitis, autoimmune hepatitis and sclerosing cholangitis, was conducted (TRANSREG) (65). All 46 enrolled patients subcutaneously received 1 million IU of aldesleukin per day for 5 consecutive days, followed by fortnightly injections of 1 million IU of aldesleukin for a total duration of 6 months. Low-dose IL-2 therapy was very well tolerated independent of the underlying disease or concomitant treatment. Immunological analyses demonstrated selective expansion and activation of the CD4+FoxP3+CD127loCD25hi Treg population without the induction of effector T cell activation in all treated patients. In parallel preliminary signals for the clinical efficacy of low-dose IL-2 therapy could be obtained during this trial.

Brief reports from open-label, therapy-controlled, single-center studies in patients with primary Sjogren’s syndrome (66), polymyositis/dermatomyositis (67) and psoriatic arthritis (uncontrolled) (68) have been published more recently by the same group. 99, 31 and 22 patients, respectively, were treated with a short-term regimen of low-dose IL-2 therapy consisting of one 5-day cycle with daily injections of 0.5 million IU of IL-2. In all three studies, increases in the Treg population were accompanied by decreases in the ratio of T_H_17 cells/Treg. Despite the short duration of treatment, more pronounced decreases in myositis-associated laboratory parameters and VAS in the IL-2 group suggested clinical responsiveness in patients with polymyositis and dermatomyositis. In psoriatic arthritis, a rapid decrease in joint symptoms and arthritis scores was observed. In Sjögren’s syndrome, the dose of glucocorticoids and immunosuppressive therapies could be reduced during the follow-up period, yet no significant difference in disease activity measures between the IL-2 and control group could be detected after the short treatment period.

More recently, a randomized, placebo-controlled phase IIa trial was conducted in patients with amyotrophic lateral sclerosis (ALS) to evaluate the therapeutic effect of three 5-day cycles of low-dose IL-2 therapy (69). The rationale was based on previous studies in ALS patients showing that decreased levels of Treg correlated with disease severity and were predictive of disease progression and survival (73). Despite a significant biological response by means of an increase in Treg, indicators for disease progression like ALSFRS-R score and plasma NFL-MSD levels did not differ significantly between the treatment and placebo group. The lack of clinical efficacy could have been due to the short treatment duration in a rather slowly progressing disease.

Several studies are currently conducted to investigate the efficacy and safety of low-dose IL-2 therapy in various other autoimmune diseases such as Crohn’s disease (NCT04263831), Behcet’s disease (NCT04065672), macrophage activation syndrome (NCT02569463), relapsing polychondritis (NCT04077736) or multiple sclerosis (NCT02424396). In addition, several modified IL-2 analogues, so called IL-2 muteins, which have either a higher selectivity for Treg or a longer *in vivo* half-life have been developed and are currently tested in phase 1 trials.

### Safety Aspects

Low-dose IL-2 therapy is generally well tolerated at the lower dose ranges up to single doses of 1.5 million IU and treatment-related AEs are usually mild and transient. The by far most frequently reported AEs are mild injection site reactions, followed by myalgia, arthralgia, fever and flu-like symptoms, which can be easily managed by symptomatic therapies or antipyretics. However, AEs occurred more frequently and had a higher severity grade when higher doses were administered (e.g. single doses of 3.0 to 4.5 million IU). Apart from mild to moderate increases in the numbers of eosinophils and NK cells and the induction of a transient and clinically negligible acute-phase reaction, no relevant deviations in the safety laboratory assessments were reported so far. By contrast to high-dose IL-2 therapy, the induction of antibodies against IL-2 has not been observed in the low-dose setting until now. Nevertheless, due to its pleiotropy, IL-2, even applied in low doses, may activate also potentially harmful cells, which bears the risk to induce or worsen autoimmunity. However, with the exception of type-1 diabetes in one clinical study (50), in none of the conducted trials so far, where various doses and treatment regimens in different diseases where tested, exacerbation of pre-existing or induction of new autoimmune syndromes was observed. Thus, based on the safety data from meanwhile numerous conducted studies in a large variety of diseases, low-dose IL-2 therapy generally can be considered a very safe therapeutic approach.

## Summary and Perspective

Data from several pilot studies and clinical trials, including first randomized trials, broadly and reproducibly prove that low-dose IL-2 therapy is very safe and capable to selectively expand a functionally competent Treg population independent of the underlying disease. In addition, these trials provided preliminary evidence for the clinical efficacy of low-dose IL-2-therapy in a large variety of inflammatory and autoimmune diseases. Low-dose IL-2 therapy therefore can be considered a novel targeted treatment option with a potentially broad applicability in various autoimmune, inflammatory and rheumatic diseases. Variations in the clinical responsiveness between different diseases or subgroups of patients could be due to a differing nature of Treg defects or the extent of their contribution to disease pathogenesis. Possibly, but probably less likely because of the quite universal response pattern to low-dose IL-2 therapy reported so far, disease-related alterations in IL-2 signaling pathways and associated molecules leading to differences in the biological responsiveness to IL-2 therapy could also affect clinical efficacy. Despite some heterogeneity in the clinical responsiveness, which also arises from quite substantial variations in study design, applied treatment regimens and treatment duration, the results of most of these trials justify the further exploration of this novel therapeutic approach in autoimmune and rheumatic diseases and provide a valuable scientific basis for placebo-controlled and larger confirmatory clinical trials. The identification of molecular, cellular and epigenetic key events in response to low-dose IL-2 therapy at a common and disease-specific level, and of biomarkers which can predict the biological and clinical responsiveness to low-dose IL-2 therapy by advanced immunophenotyping technologies will allow to select appropriate diseases or patient subgroups and to stratify patients according to their individual immune signatures in the future (74). The clinical introduction of modified formulations of IL-2 with a longer half-life or increased selectivity for Treg could further contribute to a sustained clinical and biological efficacy, including stability of Treg lineage and function, and will ease its applicability for patients. Apart from this, because of its very good safety profile and its unique mode of action, low-dose IL-2 therapy appears to be an optimal candidate for a combination therapy, e.g. with agents that can block the activity of inflammatory cytokines and pathways, which can also promote resistance of Tcon to Treg-mediated suppression, or with B cell directed therapies.

## Author Contributions

All listed authors contributed to data collection, interpretation, and critical review of the manuscript. All authors are accountable for the accuracy and integrity of the report and approved the manuscript for submission. All authors contributed to the article and approved the submitted version.

## Funding

This work was supported by German Research Foundation grants EXC2167.

## Conflict of Interest

GR and JH are members of the steering committee for a currently ongoing multi-center phase 2 clinical trial on low-dose IL-2 therapy in SLE (LUPIL-2) sponsored by ILTOO Pharma.

The remaining authors declare that the research was conducted in the absence of any commercial or financial relationships that could be construed as a potential conflict of interest.
